# Supplementary material for: The genome sequence of a weevil, Hypera rumicis (Linnaeus, 1758) (Coleoptera: Curculionidae)
Source: Wellcome Open Res. 2026 Sep 24;11:744. [Version 1] doi: 10.12688/wellcomeopenres.27925.1 (PMC13675620; doi:10.12688/wellcomeopenres.27925.1)
Supplement: Peer review history

# Reviewer report: Reviewer response for version 1

**DOI:** 10.21956/wellcomeopenres.30725.r170602

**Published:** 2026-10-16

**Authors:** Ian M Carr

**Affiliations:** 1 University of Leeds, Leeds, UK

The report describes the sequencing of the *Hypera rumicis  *genome. The data was produced in accordance with the Tree of Life’s standard protocols. The reported genome appears to be high quality and worthy of publication. The introduction is clear and easy to follow and presents no issues. The report’s results and materials and methods follow the Tree of Life’s standard text and also present no issues.

Are the datasets clearly presented in a usable and accessible format, and the assembly and annotation available in an appropriate subject-specific repository?

Yes

Are sufficient details of the sequencing and extraction, software used, and materials provided to allow replication by others?

Yes

Are the rationale for sequencing the genome and the species significance clearly described?

Yes

Are the protocols appropriate and is the work technically sound?

Yes

Reviewer Expertise:

NGS, eDNA and genome assembly

I confirm that I have read this submission and believe that I have an appropriate level of expertise to confirm that it is of an acceptable scientific standard.

# Reviewer report: Reviewer response for version 1

**DOI:** 10.21956/wellcomeopenres.30725.r170603

**Published:** 2026-09-30

**Authors:** Rajib Deb

**Affiliations:** 1 ICAR-National Research Centre, Guwahat, Assam, India

My concerns for improving the manuscript as below

 a. The genome assembly mentioned, is technically well supported by PacBio HiFi sequencing combined with Hi-C scaffolding and Hifiasm-based haplotype phasing. However, the individual haplotype k-mer completeness values (74.28% and 71.77%) are substantially lower than the combined value and should be explained, particularly in relation to the expected heterozygosity and the treatment of shared versus haplotype-specific k-mers- explanation is needed

 b. BUSCO completeness is high for haplotype 1 (98.0%) but comparatively lower for haplotype 2 (92.8%). The authors should clarify whether the missing or fragmented BUSCOs in haplotype 2 are attributable to genuine haplotype-specific sequence differences, assembly gaps, or unresolved repetitive regions.

 c. The manuscript would benefit from providing the complete assembly statistics in the main text or table, including total sequence length, number of contigs/scaffolds, contig N50, scaffold N50, L50, longest scaffold, and number/percentage of gaps.

 d. Some values in Table 4 are truncated in the presented text and therefore are difficult to evaluate.

 e. The use of Hi-C data, YaHS scaffolding, BlobToolKit assessment, contamination screening and manual curation provides a comprehensive assembly-validation framework. Nevertheless, the authors should provide a brief quantitative summary of the changes resulting from manual curation and indicate whether any potential misassemblies remain after curation.

Are the datasets clearly presented in a usable and accessible format, and the assembly and annotation available in an appropriate subject-specific repository?

Yes

Are sufficient details of the sequencing and extraction, software used, and materials provided to allow replication by others?

Yes

Are the rationale for sequencing the genome and the species significance clearly described?

Yes

Are the protocols appropriate and is the work technically sound?

Partly

Reviewer Expertise:

Genomics

I confirm that I have read this submission and believe that I have an appropriate level of expertise to confirm that it is of an acceptable scientific standard, however I have significant reservations, as outlined above.

# Reviewer report: Reviewer response for version 1

**Published:** 2026-09-24

**Authors:** Automated Benchmarking

**Affiliations:** 1 Wellcome Sanger Institute, Hinxton, UK

As this article is a Genome Note, Automated Benchmarking has been used to determine whether the reported assembly passes four defined checks established by the Earth BioGenome Project: the raw and assembled data are publicly available, the Earth BioGenome Project three-part metric is met, the k-mer completeness exceeds 90%, and the BUSCO score passes the stated threshold. For Hypera rumicis (icHypRumi1), all four checks passed. Data available: pass. ENA study PRJEB80969, raw-read accessions ERR13800505 and ERR13802647, and assembly accessions GCA_965643735.1 and GCA_965643715.1 are public. Earth BioGenome Project three-part metric: pass. Haplotype 1 is reported as 7.C.Q66, meeting the 6.C.Q40 reference standard. k-mer completeness: pass. The combined haplotype assemblies have 98.69% k-mer completeness, exceeding the Earth BioGenome Project target of 90%. BUSCO: pass. Haplotype 1, which is proposed as the reference assembly for this species, has 98.0% complete BUSCOs (96.9% single-copy and 1.1% duplicated). BUSCO software version: 5.8.3; lineage dataset: endopterygota_odb10. It meets the target of greater than 90% complete and single-copy BUSCOs. This decision uses values reported in the Genome Note and public accession records.

Reviewer Expertise:

NA

I confirm that I have read this submission and believe that I have an appropriate level of expertise to confirm that it is of an acceptable scientific standard.
